# Genetic diversity of high-elevation populations of an endangered medicinal plant

**DOI:** 10.1093/aobpla/plu076

**Published:** 2014-11-21

**Authors:** Akshay Nag, Paramvir Singh Ahuja, Ram Kumar Sharma

**Affiliations:** 1 Biotechnology Division, CSIR—Institute of Himalayan Bioresource Technology, Post Box 6, Palampur, 176061 Himachal Pradesh, India; 2 Academy for Scientific and Innovative Research (AcSIR), CSIR—Institute of Himalayan Bioresource Technology, Post Box 6, Palampur, 176061 Himachal Pradesh, India

**Keywords:** AMOVA, amplified fragment length polymorphism (AFLP), Baker's rule, genetic structure, Indian Himalayas, *Podophyllum hexandrum*, self-pollination.

## Abstract

Intraspecific genetic variation in natural populations governs their potential to overcome challenging ecological and environmental conditions. In addition, knowledge of this variation is critical for the conservation and management of endangered plant taxa. Found in the Himalayas, *Podophyllum hexandrum* is an endangered high-elevation plant species that has great medicinal importance. Here we report on the genetic diversity analysis of 24 *P. hexandrum* populations (209 individuals), representing the whole of the Indian Himalayas. In the present study, seven amplified fragment length polymorphism (AFLP) primer pairs generated 1677 fragments, of which 866 were found to be polymorphic. Neighbour joining clustering, principal coordinate analysis and STRUCTURE analysis clustered 209 individuals from 24 populations of the Indian Himalayan mountains into two major groups with a significant amount of gene flow (*N*_m_ = 2.13) and moderate genetic differentiation *F*_st_(0.196), *G′*_st_(0.20). This suggests that, regardless of geographical location, all of the populations from the Indian Himalayas are intermixed and are composed broadly of two types of genetic populations. High variance partitioned within populations (80 %) suggests that most of the diversity is restricted to the within-population level. These results suggest two possibilities about the ancient population structure of *P. hexandrum*: either all of the populations in the geographical region of the Indian Himalayas are remnants of a once-widespread ancient population, or they originated from two types of genetic populations, which coexisted a long time ago, but subsequently separated as a result of long-distance dispersal and natural selection. High variance partitioned within the populations indicates that these populations have evolved in response to their respective environments over time, but low levels of heterozygosity suggest the presence of historical population bottlenecks.

## Abstract (precis)

Despite its wide distribution across the entire Himalayan range, the current status of *Podophyllum hexandrum*, a highly important anti-cancerous herb, remains endangered. Genetic diversity characterization of 24 populations comprising of 209 individuals representing the whole of the Indian Himalayas revealed that regardless of geographic location, all of the populations are intermixed and are composed broadly of two types of genetic populations. Our findings also suggested that these populations have evolved well in response to the environment. This study will help in the formulation of conservation programs for *P. hexandrum* populations in this region.

## Introduction

Throughout history, one of the many ways in which humans have benefited from plant diversity is as a source of traditional medicines. According to the World Health Organization (WHO), as many as 80 % of the world's populations depend on traditional medicine for their primary health-care needs (WHO 1993). Most traditional therapy involves the use of plant extracts or their active principles. In the present era, unprecedented growth in global population has led to subsequent increase in human demands and overexploitation of the earth's plant resources (Gadgil and Meher-Homji 1986; Işik 2011). Most plausible scenarios today suggest that we are likely to lose a large part of our traditional wealth of medicinal plants in the near future if critical steps are not taken to conserve them (Ehrlich and Daily 1993; Badola and Aitken 2003). Currently, large numbers of medicinally important plant resources face serious threat of extinction and severe genetic loss, but detailed information is lacking. For most of these endangered medicinal plant species, effective conservation plans are minimal and very little material is available in genebanks. Further, a major emphasis on discovering new drug molecules from plant resources has contributed to the loss of natural genetic resources. Nearly 25 % of the estimated 250 000 species of vascular plants in the world may become extinct within the next 40 years, if proper conservation measures are not undertaken (Kala 2000).

Knowledge of genetic variation within species, coupled with information about their reproductive biology, is very important when establishing any conservation and management programme (Newton *et al.* 1999; Juan *et al.* 2000; Frankham 2003; Silva *et al.* 2011) aimed at preserving genetic variation within and among populations (Eriksson 2001; Silva *et al.* 2011). Knowledge of genetic diversity patterns is also important in understanding the evolutionary history of a species and in the assessment of future risks to diversity (Neel and Ellstrand 2003). With regard to endangered species, measuring genetic variation among different populations is important for prioritization of sites and management choices for future conservation programmes. For example, greatly diverse or differentiated populations could be targeted for conservation, while genetically penurious populations might be targeted for management plans to restore diversity (Godt *et al.* 1996; Petit *et al.* 1998).

In the present study, we quantified the patterns of genetic diversity within *Podophyllum hexandrum*, an endangered plant species of great medicinal importance. Using amplified fragment length polymorphism (AFLP) markers, we have examined 209 individuals of 24 natural populations of *P. hexandrum*, representing the wider geographical area of the entire Indian Himalayas ranging from the states of Jammu and Kashmir, and including the Zanskar region, Himachal Pradesh, Uttarakhand to Sikkim. The genetic diversity of *P. hexandrum* has not been studied for the entire of the Indian Himalayas, and such a large geographical area with a greater number of samples has been analysed for the first time. The present study will comprehensively reveal the overall genetic diversity prevailing in these populations and will also aid in understanding the genetic dynamics of the species. Further, this will also throw light on how these populations are persisting despite their having a small chromosome number and self-pollinating reproductive behaviour.

## Methods

### Study species

*Podophyllum hexandrum* (Himalayan mayapple; syn: *Sinopodophyllum hexandrum*, *Podophyllum emodi*) is a species of great medicinal importance. It is confined to the alpine regions of Afghanistan, Pakistan, Nepal, Bhutan, South West China and India (Airi *et al.* 1997; Choudhary *et al.* 1998). Despite its wider distribution in the entire Indian Himalayan range, from Ladakh to Sikkim at an elevation of 3000–4200 m, the current status of *P. hexandrum* is now endangered. The rhizomes and roots of *P. hexandrum* contain anti-tumour lignans such as podophyllotoxin, 4′-dimethyl podophyllotoxin and podophyllotoxin 4-*O*-glucoside (Tyler *et al.* 1988; Broomhead and Dewick 1990). Among these lignans, podophyllotoxin or podophylloresin is most important for its use in the semi-synthesis of anti-cancer drugs etoposide and teniposide (Issell *et al.* 1984; Canel *et al.* 2000). Podophyllotoxin acts as an inhibitor of microtubule assembly. These drugs are used in the treatment of lung cancer, testicular cancer, neuroblastoma, hepatoma and other tumours. It also shows antiviral activities by interfering with some critical viral processes (Giri and Narasu 2000). The podophyllotoxin content of Himalayan mayapple is quite high (4.3 %) compared with other species of *Podophyllum*, notably *Pelargonium peltatum* (0.25 %), the most common species in the American subcontinent (Jackson and Dewick 1984). However, the percentage of resin varies greatly at different growth phases, with age of the plant, seasonal variation and different geographical sites (Purohit *et al.* 1999).

The life cycle of *P. hexandrum* is 5–6 years. Flowers blossom before the leaves grow out. According to the latest report, occasional cross-pollination has been observed in *P. hexandrum* (Xiong *et al.* 2013); however, its morphological and biological characteristics are adapted to self-pollination and effective sexual reproduction. The self-pollination mechanism of the plant is very unique. When the flower is under blossom or just in blossom, the position of the gynoecium is upright; however, when it reaches full blossom stage, the gynoecium takes a full turn and because of this, the entire gynoecium gets closer to an anther to become pollinated. Fruit bearing is almost 100 %. Thus it appears the plant shows considerable fitness (Shaobin *et al.* 1997; Xu *et al.* 1997). The important point here is that most of the members of Berberidaceae are cross-pollinated including *P. peltatum*, the North American counterpart of Himalayan Mayapple. The disjunction between the two species is estimated to have happened ∼6.52 ± 1.89 million years ago (Liu *et al.* 2002). Lack of pollinators and the nectarless character (Crants 2008) of the flower might have been responsible for the evolution of self-pollination in this plant.

In natural conditions, the dispersal of seeds is facilitated primarily by herbivores, mainly Himalayan grazers that travel great distances; hence the seed dispersal distance of *P. hexandrum* is reasonably good (Rajkumar and Ahuja 2010). *Podophyllum hexandrum* has a wide region of distribution; however, within that region, it appears primarily in valleys with secondary vegetation. In any given population, the plant shows a clumping distribution pattern (Ma and Hu 1996).

Traditionally, *P. hexandrum* has been used in folk medicine in small quantities by local healers as a cure for ulcers, cuts, wounds and skin diseases (Negi *et al.* 2011), but commercialization of this plant in recent years has increased the demand and consequent exploitation of the species. Owing to habitat fragmentation (Young *et al.* 1996), overexploitation, long dormancy, low rate of natural regeneration and overgrazing, it has been classified as an endangered species (Kala 2005). There is an urgent need to conserve the genetic diversity of this prized medicinal plant, which may become extinct if its reckless exploitation continues. Earlier studies of genetic diversity in Himalayan populations have been restricted to a relatively limited geographic area (Xiao *et al.* 2006*a*, *b*; Alam *et al.* 2008; Naik *et al.* 2010; Li *et al.* 2011). As has been suggested by its pollination mechanism (namely, self-pollination), populations are expected to be genetically structured. Further, owing to a small chromosome number (2*n* = 12) (Nag and Rajkumar 2011) with a very large genome (*C* value = 16.075 Gb) (Nag *et al.* 2011), *P. hexandrum* might be experiencing severe evolutionary pressure against adaptation as suggested by the large genome size constraint hypothesis (Knight *et al.* 2005).

### Plant materials

**Figure 1.**
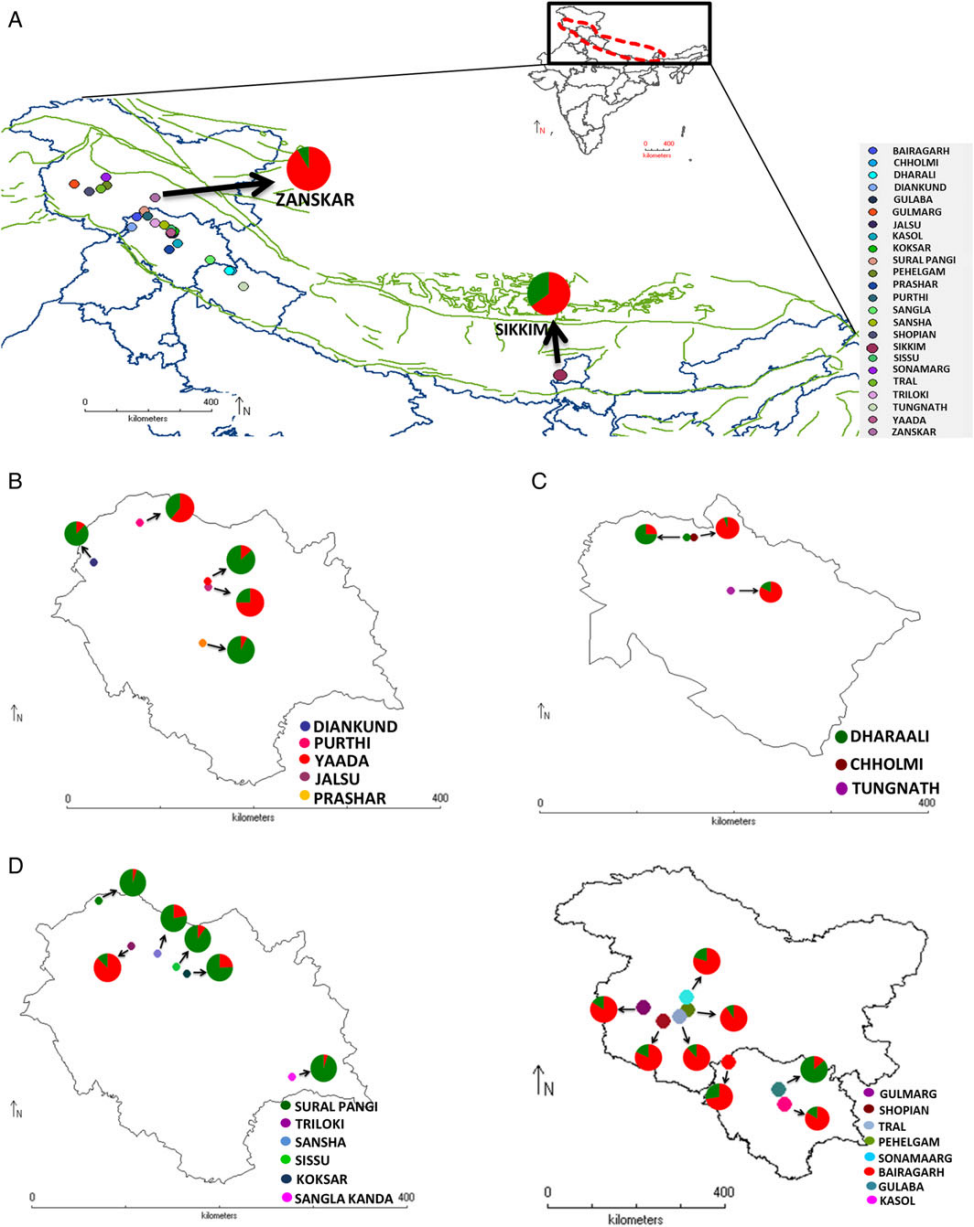
Geographic distribution of sampled populations of *P. hexandrum* from the Indian Himalayas with pie charts representing the percentage of the two genetic pools from each of the populations. (A) Map representing all the sampled locations, (B) Dhauladhar range, (C) Shivalik/Garhwal Himalayas, (D) Greater Himalayas, (E) Pir Panjal range.

A total of 24 different geographical locations, ranging from Kashmir to the Sikkim Himalayas and representing most of the Indian Himalayas, were visited for sample collection in the present study during 2008 and 2012 (Table 1, Fig. 1). The Himalayan mountain ranges included were the Dhauladhar range, the Pir Panjal range, the Shivalik/Garhwal range, the Greater Himalayan range, the Zanskar range and the Kangchenjunga Himal section. The Zanskar range, which is a Trans-Himalayan range, and the Kangchenjunga Himal section are subranges of the Greater Himalayas. Young leaves of the Himalayan mayapple were collected in silica gel by changing the gel periodically, until the sample was completely dried. The minimum distance between sample plants within a population was kept at ∼5 m. The extent of exploitation of the plant is such that at some locations, the number of plants per quadrat (1 m × 1 m) was 0.6 and hence we kept 5 as the minimum sample size in the study; however, we sampled up to 25 plants per population. The total number of samples collected was 224, of which 209 were chosen for analysis based on the presence of good-quality DNA profiles.

**Table 1.** Details of the locations from where samples of *P. hexandrum* were collected for this study. *N*, number of individuals in a population.

Location	Himalayan range	State	Altitude (in metres MSL)	Geographic coordinates		*N*
				Latitude	Longitude	
Bairagarh	Pir Panjal	Himachal Pradesh	2292	32.9064	76.1616	9
Chholmi	Garhwal Himalaya	Uttarakhand	2899	31.0269	78.8704	5
Dharali	Garhwal Himalaya	Uttarakhand	3005	31.0283	78.7983	12
Diankund	Dhauladhar	Himachal Pradesh	2154	32.5417	76.0275	13
Gulaba	Pir Panjal	Himachal Pradesh	2994	32.3188	77.2035	12
Gulmarg	Pir Panjal	Jammu and Kashmir	2156	34.0614	74.3876	5
Jalsu	Dhauladhar	Himachal Pradesh	3359	32.3063	77.1429	10
Kasol	Pir Panjal	Himachal Pradesh	2770	31.9919	77.3392	13
Koksar	Greater Himalayas	Himachal Pradesh	3136	32.4138	77.2349	17
Pehelgam	Pir Panjal	Jammu and Kashmir	2218	34.0149	75.3106	5
Prashar	Dhauladhar	Himachal Pradesh	2315	31.7644	77.0897	16
Purthi	Dhauladhar	Himachal Pradesh	2978	32.9225	76.474	5
Sangla Kanda	Greater Himalayas	Himachal Pradesh	2915	31.4201	78.2574	5
Sansha	Greater Himalayas	Himachal Pradesh	3273	32.6125	76.9493	5
Shopian	Pir Panjal	Jammu and Kashmir	2182	33.785	74.7942	5
Sikkim	Greater Himalayas (Kangchenjunga Himal section)	Sikkim	2741	27.3807	88.2551	5
Sissu	Greater Himalayas	Himachal Pradesh	3127	32.4833	77.1299	7
Sonamarg	Pir Panjal	Jammu and Kashmir	3031	34.2954	75.2919	5
Sural Pangi	Greater Himalayas	Himachal Pradesh	2715	33.1209	76.3788	5
Tral	Pir Panjal	Jammu and Kashmir	2433	33.8787	75.1356	5
Trilokinath	Greater Himalayas	Himachal Pradesh	2910	32.6837	76.6962	9
Tungnath	Garhwal Himalaya	Uttarakhand	3448	30.4883	79.2162	5
Zanskar	Greater Himalaya	Jammu and Kashmir	3810	33.587	76.696	25
Dharali	Garhwal Himalaya	Uttarakhand	3005	31.0283	78.7983	12

### DNA isolation and molecular analysis

Total genomic DNA was isolated following the CTAB method (Doyle and Doyle 1987; Doyle 1990) with minor modifications. Deoxyribonucleic acid concentrations were determined using a Nanodrop spectrophotometer (Thermo Scientific) followed by a quality check on ethidium bromide-stained agarose gels, using known amounts of uncut λ DNA as a standard.

The AFLP protocol was carried out following the procedure described by Vos *et al.* (1995) with minor modifications. Genomic DNA (250 ng) was restricted with *Eco*RI/*Mse*I enzyme mix and ligated to standard adapters using the T4 DNA ligase. The adapter-ligated DNA served as a template for preamplification, with PCR parameters of 20 cycles at 94 °C for 30 s, 56 °C for 1 min and 72 °C for 1 min. After screening 36 primer pairs for four individuals from four populations, seven primer pairs (Table 2) were chosen for the full survey because they resulted in clear and reproducible bands **[see Supporting Information]**. Selective amplification was carried out with 2.5 μL of these diluted products using *Eco*RI primers (fluorescently labelled with NED, FAM and JOE) and *Mse*I primers, Taq polymerase, PCR buffer, MgCl_2_, each dNTPs and deionized water in a final volume of 10 μL. The first selective amplification cycle consisted of 94 °C for 30 s, 65 °C for 30 s and 72 °C for 1 min. The annealing temperature was lowered by 0.7 °C per cycle during the next 12 cycles, followed by 23 cycles at 94 °C for 30 s, 56 °C for 30 s and 72 °C for 1 min. All PCRs were performed on the i-cycler PCR system (Bio-Rad, Australia). 0.5 μL of each selective PCR product was mixed with 0.3 μL of Gene Scan-500 ROX size standard (Applied Biosystems) and 9.2 μL of highly deionized formamide. This mixture was denatured at 94 °C for 5 min, followed by immediate chilling on ice and these denatured products were loaded on an ABI 3730xl automated DNA Analyser (Applied Biosystems, Hitachi) to visualize the amplified fragments.

**Table 2.** List of AFLP primer pairs used in the study.

S. no.	Primer combination	No. of bands	No. of polymorphic bands	Percentage polymorphism (%)
1	E-ACA + M-CTGC	325	135	41.53
2	E-AAC + M-CAG	256	92	35.93
3	E-AAG + M-CTAG	166	128	77.10
4	E-ACC + M-CAT	257	103	40.07
5	E-ACT + M-CAG	229	126	55.02
6	E-ACC + M-CAG	261	154	59.03
7	E-AGG + M-CTT	183	128	69.94

The software program GeneMapper 3.7 (Applied Biosystems) was used to analyse electropherograms generated by automated genotyping using the ABI 3730xl automated DNA analyser. The large amount of data generated by the automated DNA analyser was checked manually a number of times to exclude unreliable detection and to improve the quality of data. The size range of amplified fragments, peak height threshold in terms of relative fluorescence units (rfu) and bandwidth were considered to be the most important scoring parameters; different sets of parameters were tested, and the parameter set that was optimized for the best fit was used for our analyses. Amplified fragments of 50–500 base pairs having present (1) and absent (0) peaks were extracted using GeneMapper 3.7. The resulting binary matrix was exported in the form of comma-separated text for data analysis.

### Data analysis

Calculations for genetic distance, pairwise population matrix of Nei's genetic identity, allele frequency by population, Mantel test for correlation of genetic and geographic distance and principal coordinate analysis (PCoA) were conducted using GenAlEx 6.501 (Peakall and Smouse 2006, 2012). STRUCTURE version 2.3.4 (Pritchard *et al.* 2000) was used to infer the genetic structure so as to obtain an estimate of the likely number of population genetic clusters (*K*). The numbers of clusters of the populations (*K*) were identified by performing six iterations and setting the value of *K* from 1 to 25 with a burn-in period of 100 000 and 100 000 number of the Markov Chain Monte Carlo (MCMC) repeats after burn-in. The maximal value of LnP(D), the posterior probability of data as per Evanno *et al.* (2005), was obtained using STRUCTURE HARVESTER (Earl and vonHoldt 2012). To further confirm the number of genetic clusters, the value of *K* was estimated through analysis of molecular variance (AMOVA)-based clustering using kMeans software (Meirmans 2012). To infer the partitioning of the diversity, *G*_st_ & *G′*_st_ software package GenoType/GenoDive (Meirmans and Van Tienderen 2004) was used. In the first step GenoType detects the genotyping errors and prepares an input file for GenoDive. In the second step GenoDive calculates the parameters of diversity and diversity partitioning. AFLPSURV (Vekemans *et al.* 2002), which follows a Bayesian method with non-uniform prior distribution (Zhivotovosky 1999), was used to infer the genetic relationships among populations by calculating Nei's unbiased genetic distance (Lynch and Milligan 1994) among all possible pairs of populations from allele frequencies. Hierarchical AMOVA and *F*_st_ was conducted using ARLEQUIN 3.5.1.2 (Excoffier and Lischer 2010). The input file for ARLEQUIN was prepared using the program CONVERT (Glaubitz 2004). The dendrogram was computed by using the neighbour joining (NJ) clustering with DARwin5 version 5.0.158 (Perrier and Jacquemoud-Collet 2006).

## Results

### AFLP analysis and polymorphism

Scoring the sampled material of *P. hexandrum* (209 individuals, 24 populations) for seven AFLP primer combinations resulted in 1677 unambiguous fragments in the size range of 50–500 bp, of which 866 (51.65 %) were polymorphic (Table 2). The mean number of fragments per individual was found to be 105.5. The maximum number of polymorphic bands was found in the Zanskar population (40.42 %), followed by the Koksar population (37.99 %), whereas the Chholmi population had the minimum number of polymorphic bands (7.27 %). The number of private alleles in each population ranged from 0 to 40, and comprised 25.08 % of the total bands. The population of Zanskar contained a maximum 40 private alleles, followed by the Triloki population (28 private alleles), whereas the Tral population had only one private allele. We did not find any private alleles in the Pehelgam, Chholmi, Sural Pangi and Sansha populations. Overall heterozygosity (Nei's unbiased diversity, u*h*) estimates recorded in the AFLP analysis were found to be very low, with the maximum observed heterozygosity (0.155 ± 0.008) in the Eastern Himalayan population collected from Sikkim and the lowest heterozygosity in the Sural Pangi population (0.043 ± 0.005). Shannon's information index (*I*) values also complemented these findings (Table 3).

**Table 3.** Population genetic parameters of the 24 populations comprising 209 individuals of *Podophyllum hexandrum* from the Indian Himalayas. *N*, number of individuals in a population; u*h*, Nei's unbiased diversity; *I*, Shannon's information index.

Location	N	No. of polymorphic alleles	No. of private alleles	% Polymorphism	u*h*	*I*
Bairagarh	9	288	9	33.3	0.122 ± 0.006	0.166 ± 0.008
Chholmi	5	75	0	8.7	0.052 ± 0.006	0.058 ± 0.006
Dharali	12	247	10	28.5	0.087 ± 0.006	0.124 ± 0.008
Diankund	13	284	12	32.8	0.093 ± 0.005	0.137 ± 0.007
Gulaba	12	227	7	26.2	0.079 ± 0.005	0.113 ± 0.007
Gulmarg	5	187	3	21.6	0.118 ± 0.008	0.135 ± 0.009
Jalsu	10	281	13	32.4	0.111 ± 0.006	0.154 ± 0.008
Kasol	13	319	12	36.8	0.107 ± 0.006	0.156 ± 0.008
Koksar	17	331	24	38.2	0.102 ± 0.005	0.154 ± 0.008
Pehelgam	5	145	0	16.7	0.086 ± 0.007	0.098 ± 0.008
Prashar	16	290	7	33.5	0.084 ± 0.005	0.127 ± 0.007
Purthi	5	272	6	31.4	0.148 ± 0.008	0.176 ± 0.009
Sangla Kanda	5	196	8	22.6	0.082 ± 0.006	0.097 ± 0.007
Sansha	5	170	0	19.6	0.087 ± 0.007	0.103 ± 0.008
Shopian	5	205	1	23.7	0.091 ± 0.007	0.104 ± 0.008
Sikkim	5	298	17	34.4	0.155 ± 0.008	0.185 ± 0.009
Sissu	7	206	3	23.8	0.080 ± 0.006	0.103 ± 0.007
Sonamarg	5	194	2	22.4	0.085 ± 0.007	0.099 ± 0.008
Sural Pangi	5	127	0	14.7	0.043 ± 0.005	0.048 ± 0.006
Tral	5	179	2	20.7	0.108 ± 0.008	0.122 ± 0.009
Trilokinath	9	290	28	33.5	0.123 ± 0.007	0.167 ± 0.009
Tungnath	5	254	8	29.3	0.138 ± 0.008	0.164 ± 0.009
Yada	6	229	7	26.4	0.098 ± 0.006	0.123 ± 0.008
Zanskar	25	350	40	40.4	0.090 ± 0.005	0.142 ± 0.007

### Genetic differentiation and partitioning of populations

Overall *F*_st_ revealed in Arlequin analysis was 0.196 (Table 4). *G′*_st_ was found to be 0.20, whereas *G*_st_ = 0.19. Pairwise *F*_st_ analyses (Table 5) showed that the populations from Sural Pangi (Greater Himalayan range) and Sonamarg (Pir Panjal range) and Sural Pangi (Greater Himalayan range) and Shopian (Pir Panjal range) were found to be most divergent (*F*_st_ = 0.58) of the populations, whereas the minimum *F*_st_ (0.04) was observed between the Gulaba (Pir Panjal range) and Diankund (Dhauladhar range) populations, and the Dharali (Garhwal Himalaya) and Diankund (Dhauladhar range) populations.

**Table 4.** Table of analysis of molecular variance along with *F*_st_ value, as calculated in ARLEQUIN.

Source	Degrees of freedom	Sum of squares	Variance components	Percentage variation	*F*_st_
Among populations	23	2991.200	10.247	20	0.196
Within populations	185	7797.613	42.149	80

**Table 5.** Pairwise *F*_st_ and Nei's genetic distances between 24 populations. Values above the diagonal represent Nei's genetic distances and values below the diagonal represent *F*_st._

	1	2	3	4	5	6	7	8	9	10	11	12	13	14	15	16	17	18	19	20	21	22	23	24		
1	0	0.034	0.028	0.029	0.024	0.032	0.028	0.028	0.027	0.020	0.020	0.020	0.023	0.035	0.051	0.058	0.051	0.046	0.046	0.048	0.073	0.045	0.042	0.069	1	Bairagarh
2	0.19	0	0.037	0.057	0.046	0.040	0.047	0.045	0.031	0.043	0.055	0.045	0.052	0.050	0.058	0.072	0.059	0.060	0.060	0.075	0.092	0.058	0.057	0.076	2	Sonamrg
3	0.14	0.25	0	0.051	0.042	0.044	0.037	0.039	0.039	0.039	0.042	0.035	0.041	0.053	0.065	0.072	0.066	0.066	0.064	0.077	0.096	0.061	0.059	0.078	3	Shopian
4	0.14	0.33	0.29	0	0.029	0.042	0.043	0.046	0.043	0.025	0.025	0.031	0.032	0.049	0.063	0.061	0.060	0.055	0.055	0.052	0.075	0.055	0.053	0.078	4	Tral
5	0.1	0.27	0.24	0.14	0	0.027	0.034	0.039	0.030	0.018	0.022	0.023	0.023	0.035	0.051	0.051	0.046	0.043	0.042	0.038	0.066	0.043	0.043	0.065	5	Gulmarg
6	0.17	0.28	0.29	0.25	0.14	0	0.045	0.052	0.033	0.023	0.037	0.036	0.028	0.054	0.069	0.071	0.059	0.063	0.059	0.047	0.090	0.062	0.063	0.087	6	Pehelgam
7	0.11	0.22	0.17	0.18	0.13	0.22	0	0.031	0.035	0.034	0.032	0.025	0.035	0.040	0.049	0.054	0.048	0.046	0.045	0.061	0.074	0.044	0.041	0.058	7	Sikkim
8	0.12	0.23	0.2	0.22	0.17	0.27	0.09	0	0.037	0.042	0.038	0.032	0.043	0.044	0.053	0.059	0.054	0.049	0.051	0.069	0.078	0.047	0.047	0.066	8	Tungnath
9	0.1	0.15	0.19	0.19	0.11	0.16	0.11	0.13	0	0.029	0.034	0.027	0.037	0.036	0.043	0.054	0.045	0.042	0.044	0.052	0.073	0.040	0.044	0.058	9	Purthi
10	0.09	0.23	0.21	0.11	0.06	0.11	0.14	0.19	0.12	0	0.016	0.018	0.013	0.041	0.059	0.059	0.053	0.052	0.048	0.036	0.071	0.049	0.049	0.075	10	Triloki
11	0.1	0.31	0.24	0.13	0.11	0.22	0.15	0.19	0.17	0.08	0	0.015	0.013	0.042	0.060	0.056	0.055	0.050	0.047	0.037	0.068	0.049	0.046	0.072	11	Kasol
12	0.1	0.26	0.2	0.16	0.11	0.21	0.1	0.15	0.12	0.09	0.08	0	0.016	0.034	0.050	0.049	0.046	0.043	0.040	0.037	0.063	0.038	0.039	0.062	12	Jalsu
13	0.15	0.32	0.27	0.21	0.15	0.19	0.21	0.26	0.22	0.08	0.09	0.11	0	0.041	0.063	0.057	0.053	0.051	0.047	0.033	0.074	0.049	0.046	0.076	13	Zanskar
14	0.2	0.29	0.3	0.28	0.2	0.31	0.2	0.23	0.19	0.23	0.25	0.2	0.27	0	0.014	0.019	0.017	0.011	0.012	0.038	0.035	0.013	0.017	0.033	14	Koksar
15	0.31	0.37	0.39	0.37	0.31	0.41	0.28	0.31	0.25	0.34	0.35	0.31	0.38	0.1	0	0.012	0.014	0.011	0.010	0.056	0.033	0.010	0.019	0.024	15	Prashar
16	0.32	0.44	0.43	0.37	0.31	0.43	0.28	0.32	0.29	0.32	0.33	0.29	0.36	0.12	0.08	0	0.016	0.014	0.013	0.051	0.029	0.014	0.023	0.030	16	Sissu
17	0.26	0.35	0.38	0.33	0.25	0.35	0.22	0.27	0.22	0.27	0.3	0.26	0.32	0.09	0.09	0.09	0	0.012	0.014	0.047	0.036	0.013	0.022	0.028	17	Yada
18	0.27	0.36	0.38	0.32	0.25	0.37	0.25	0.27	0.23	0.29	0.29	0.26	0.33	0.07	0.07	0.09	0.06	0	0.007	0.044	0.029	0.008	0.018	0.027	18	Diankund
19	0.28	0.39	0.4	0.35	0.27	0.39	0.26	0.31	0.26	0.29	0.3	0.26	0.32	0.08	0.08	0.09	0.09	0.04	0	0.037	0.027	0.008	0.017	0.028	19	Gulaba
20	0.29	0.51	0.5	0.37	0.27	0.38	0.33	0.39	0.3	0.22	0.24	0.25	0.24	0.25	0.39	0.4	0.35	0.31	0.31	0	0.058	0.042	0.050	0.072	20	Chholmi
21	0.4	0.58	0.58	0.48	0.43	0.57	0.4	0.44	0.4	0.39	0.39	0.38	0.44	0.24	0.27	0.28	0.3	0.23	0.24	0.54	0	0.028	0.044	0.046	21	Sural_Pangi
22	0.27	0.36	0.37	0.33	0.26	0.38	0.24	0.27	0.23	0.28	0.3	0.24	0.32	0.08	0.07	0.09	0.08	0.04	0.05	0.32	0.23	0	0.015	0.024	22	Dharali
23	0.22	0.36	0.36	0.31	0.24	0.39	0.19	0.24	0.22	0.26	0.27	0.22	0.29	0.09	0.13	0.16	0.13	0.11	0.12	0.39	0.38	0.09	0	0.035	23	Sansha
24	0.35	0.45	0.45	0.42	0.35	0.48	0.28	0.33	0.29	0.36	0.38	0.34	0.42	0.21	0.18	0.22	0.18	0.18	0.22	0.5	0.4	0.17	0.24	0	24	Sangla Kanda

Analysis of molecular variance analysis revealed that the majority of the variance was restricted to within-population variation (80 %), whereas variance partitioned among population was 20 %. Significant gene flow (*N*_m_) was recorded between the populations. On the basis of *F*_st_[*N*_m_ = (1/*F*_st_− 1)/4], *N*_m_ was found to be 1.02, whereas the value of gene flow on the basis of Gst [*N*_m_ = *G*_st_(1 − *G*_st_)/*G*_st_] was found to be 2.13, which indicated a considerable intermixing and low genetic differentiation among populations.

### Population genetic structure and cluster analysis

**Figure 2.**
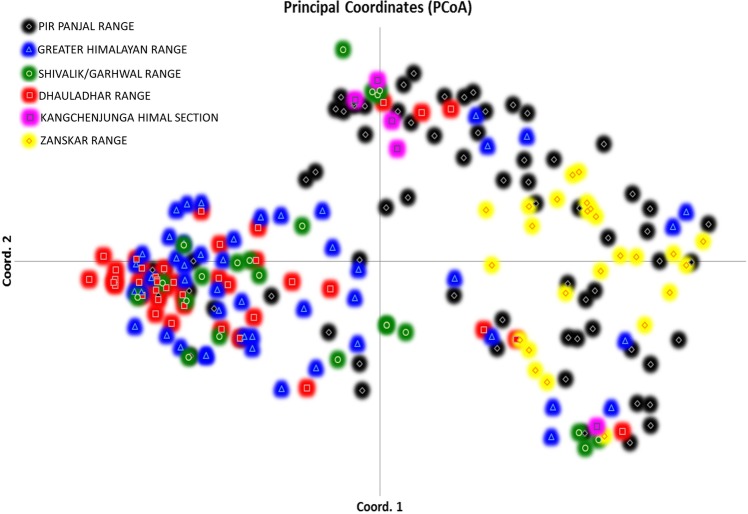
Principal coordinate analysis showing the differentiation of 209 individuals of *P. hexandrum* from 24 populations from the Indian Himalayas according to the mountain ranges.

**Figure 3.**
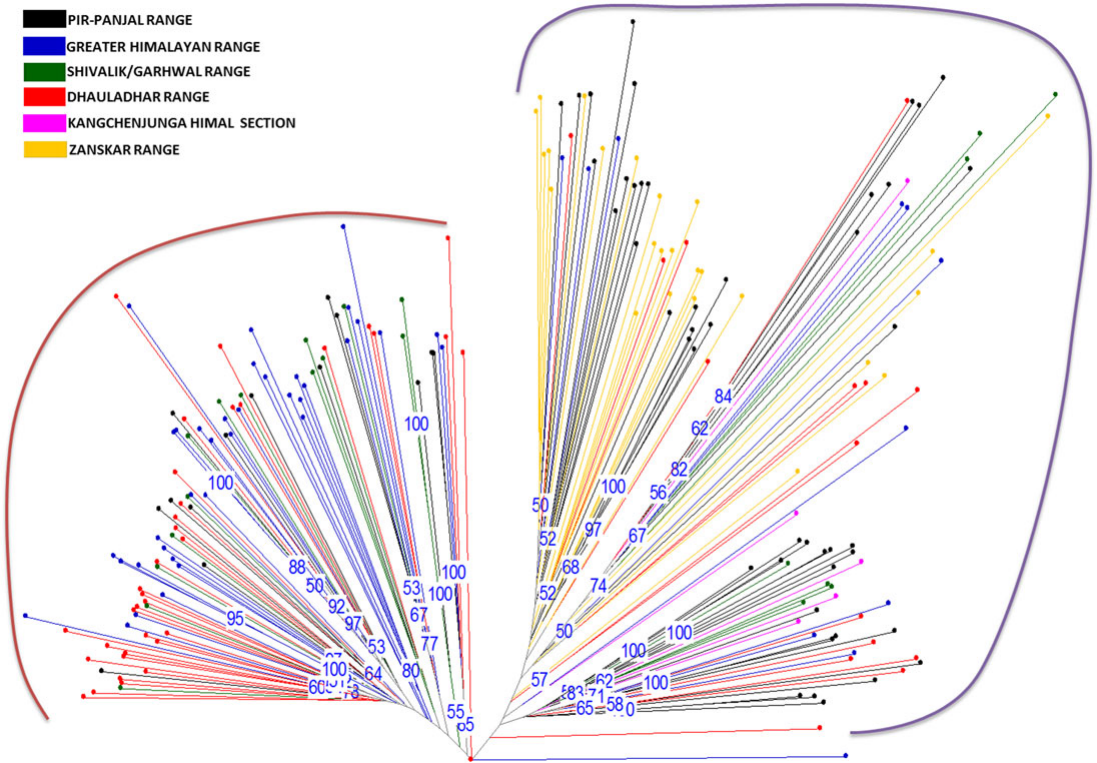
Neighbour-joining tree based on genetic distances of 209 individuals of *Podophyllum hexandrum* from the Indian Himalayas. Numbers above branches indicate bootstrap values >50 % (1000 replicates).

**Figure 4.**
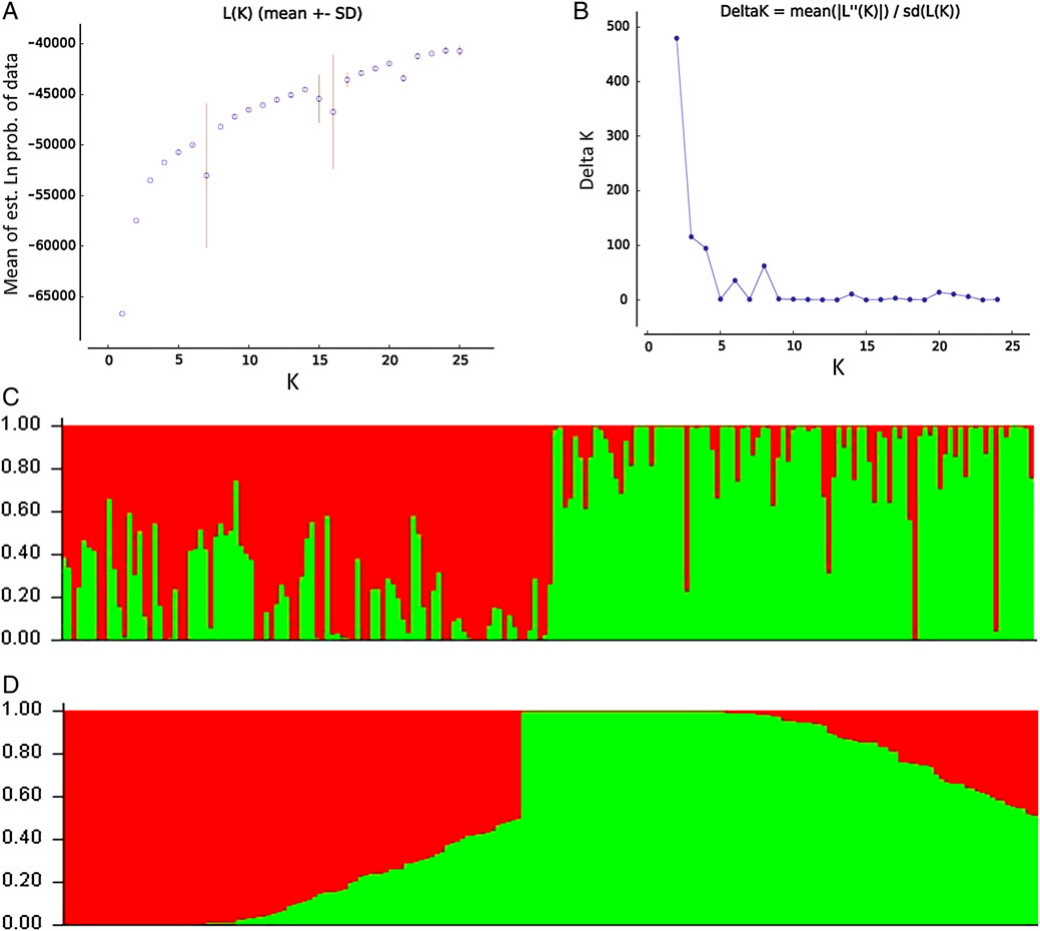
STRUCTURE inferences of *P. hexandrum* populations based on AFLP genotyping. (A) log likelihood, LnP(D), (B) changes in the log likelihood, Δ(*K*), for different number of groups. (C) Bar plots represent STRUCTURE inferences of individual assignments (*K* = 2) as inferred in the Structure Harvester web v. 0.6.93. Each vertical bar represents one individual. (D) The bar plot represents individuals arranged according to its most likely ancestry. Each colour represents the most likely ancestry of the cluster from which the genotype or partial genotype was derived.

AFLP-based genetic diversity analysis in the 24 populations of *P. hexandrum* was carried out using three different but complementary approaches, factorial analysis or PCoA, neighbour Joining (NJ)-based hierarchical clustering and Bayesian model-based clustering. Principal coordinate analysis (Fig. 2) complemented NJ (Fig. 3) cluster analysis in providing an overall view of genetic diversity in the natural populations of *P. hexandrum*. The first three axes effectively captured the entire diversity (91 %) in the *P. hexandrum* populations and revealed two major groups. A Mantel test between the genetic and geographic distances showed no correlation **[see Supporting Information]**. This was further confirmed by the AMOVA analysis between the mountain ranges, which showed that majority of variance was found within mountain ranges (91 %) **[see Supporting Information]**. The dendrogram (Fig. 3) obtained by NJ analysis showed that populations from the Zanskar and Kashmir Valleys (Gulmarg, Sonamarg, Pehelgam, Tral and Shopian) were clustered in one group along with populations from Bairagarh, Jalsu, Kasol, Triloki, Purthi, Tungnath and Sikkim (group I). The majority of the populations of the Pir Panjal range (six out of seven populations) remained together in Group I, along with two populations from the Dhauladhars and a single population each from the Zanskar, Shivalik/Garhwal, Greater Himalayas and Kangchenjunga Himal section (Table 6). The second group is composed of majority of the populations from the Greater Himalayan trail (Sansha, Koksar, Sural Pangi, Sangla Kanda and Sissu) and the Dhauladhar range (Prashar, Yada and Diankund) along with two populations from Garhwal Himalayas (Chholmi and Dharali) and a single population from Pir Panjal (Gulaba). Clustering is quite distinct with only 10 out of 209 individuals recorded as intermixing between the groups. Surprisingly, individuals from a single population did not cluster together into a single subgroup. Bayesian model-based STRUCTURE analyses showed that the maximum likelihood of clustering of the AFLP data [LnP(D)] was obtained when samples were clustered into two groups (*K* = 2). This confirms that among the populations included in the study, two types of gene pools/genetic populations are found in the Indian Himalayas (Fig. 4). The percentage of individuals with pure grouping was found to be 73.4 %, whereas 26.6 % individuals showed mixed grouping at various levels.

**Table 6.** Cluster analysis results for populations, with mountain ranges identified.

Group I	Group II
Bairagarh (Pir Panjal)	Chholmi (Shivalik or Garhwal Himalayas)
Pehelgam (Pir Panjal)	Dharali (Shivalik or Garhwal Himalayas)
Sonamarg (Pir Panjal)	
Gulmarg (Pir Panjal)	Sansha (Greater Himalayas)
Shopian (Pir Panjal)	Koksar (Greater Himalayas)
Tral (Pir Panjal)	Sangla Kanda (Greater Himalayas)
Kasol (Pir Panjal)	Sissu (Greater Himalayas)
Leh (Zanskar)	Sural Pangi (Greater Himalayas)
Jalsu (Dhauladhar)	Diankund (Dhauladhar)
Purthi (Dhauladhar)	Yada (Dhauladhar)
Tungnath (Shivalik or Garhwal Himalayas)	Prashar (Dhauladhar)
Triloki (Greater Himalayas)	Gulaba (Pir Panjal)
Sikkim (Kangchenjunga Himal section)

## Discussion

Amplified fragment length polymorphism markers have been widely used to study the genetic diversity and population structure of various endangered plant species like *Leucopogon obtectus* (Gardens *et al.* 2001), *Eryngium alpinum* (Gaudeul *et al.* 2000), *Limonium dufourii* (Palacios *et al.* 1999), *Silene tatarica* (Tero *et al.* 2003) and others. The efficiency of a DNA marker system for analysing diversity relies upon the extent of polymorphism detected by uncovering a large number of markers spanning the whole genome (Luikart *et al.* 2003). Amplified fragment length polymorphism markers are ideal for detecting polymorphisms, as the variable regions detected are based on restriction enzyme sites and thus essentially reveal through whole-genome scans even minor genetic variations within any given organism (Mueller and Wolfenbarger 1999). We were able to obtain sufficient numbers of polymorphic markers (866; 51.65 %) for the estimation of population genetic parameters, in accordance with the criteria for the critical number of dominant markers suggested by Staub *et al.* (2000); (80 bands) and Mariette *et al.* (2002); (100–200 bands) for reliable estimation of population genetic parameters. Additionally, three primer combinations having detected a large number of bands suggest the potential of these markers for future population biology studies in this species.

### Genetic differentiation

Generally, taxa with self-pollinating behaviour have the majority of variance partitioned among populations (Loveless and Hamrick 1984). This species, therefore, might be expected to have a diverse distribution of AFLP variation among populations, but the present study does not support this *a-priori* expectation in overall levels of inter-population differentiation across the 24 populations surveyed (within population 80 % and among population 20 %, with *F*_st_ = 0.196 and *G′*_st_ = 0.20). However, significant population differentiation reported previously in allozymes (Bhadula *et al.* 1996) and various DNA fingerprinting marker studies (Xiao *et al.* 2006*a*, *b*; Alam *et al.* 2008; Naik *et al.* 2010) might have resulted due to the fact that the targeted populations were locally restricted, which is also congruent with differentiation inferences in the subpopulation in the current study. Current inferences are based on sampled populations from a wide geographical range covering the whole of the Indian Himalayas. Moreover, low population size also leads to lower levels of genetic variation, which is a general trend in endangered plants (Loveless and Hamrick 1984). The number of plants of *P. hexandrum* is very small as compared with other non-endangered plants found in any of the locations we sampled. The overall genetic differentiation between the populations in this study was found to be moderate (*F*_st_ = 0.196, *G′*_st_ = 0.20), which is evident from the cluster analysis, as all the individuals from a population remain clustered in either of the two groups, confirming that genetic structure, although weak, is present in these populations. Low values of unbiased heterozygosity (u*h* = 0.043–0.155) and Shannon's information index (*I* = 0.048–0.185) suggest that population bottlenecks resulted due to small population size. This also accounts for reduced genetic variation among the sampled populations in the present study.

### Formation of Himalayas and subsequent evolution of *P. hexandrum* populations

Although self-pollinated, *P. hexandrum* is also capable of occasional cross-pollination, and this phenomenon accounts for another source of low genetic variation among populations. The seed set in the cross-pollinated plants is found to be almost the same as that in self-fertilized plants (Xiong *et al.* 2013) and it is believed that self-pollinated species are almost always derived from cross-fertilizing ancestors (Stebbins 1957; Wyatt 1988). *Podophyllum peltatum*, the species found in the North American subcontinent, is cross-pollinated (i.e. self-incompatible; SI) whereas *P. hexandrum* is self-compatible (SC). The sister relationship between the two species is well documented, and it is estimated that these species became separated ∼6.94 ± 3.94 million years ago (Liu *et al.* 2002). The disjunction between the two species coincides with the time of upsurge of the Himalayas, and it appears that the self-pollinating mode of reproduction has evolved from the cross-fertilizing *P. peltatum* and has also been proven phylogenetically (Wang *et al.* 2007). The last rapid upsurge of the Himalayas began ∼4–3 million years ago in the late Miocene. This suggests that a shift from SI to SC might have evolved as a result of this geographical development. The pollinator fauna are known to decline in terms of both species and number with rise in elevation. Thus, the uplifted habitat of the plant must have resulted in the scarcity of pollinators in early spring. In the process of evolution, the flower of *P. hexandrum* has adapted to delayed selfing, i.e. it tends to allow earlier cross-pollination to predominate when pollinators are available, which seems to be a reproductive strategy in response to the scarcity of the pollinators. Further, attractive, open, cup-shaped showy flowers with large anthers of *P. hexandrum* are characters of a cross-pollinating species. This suggests that the self-pollination mode of reproduction has evolved in this plant only to counter pollinator scarcity, although cross-pollination has not been eradicated. Self-pollination might have been responsible for the dispersal of populations across the Himalayas following Baker's rule, which suggests that following long-distance dispersal, a solitary propagule is much more likely to reproduce and generate a sexually reproducing population if it is capable of self-fertilization (Baker 1955). If the new colony thus established is well adapted to its new environment, it can spread throughout the area, where favourable conditions are found, even though its capacity for genetic variation is greatly reduced (Stebbins 1957) and some of the traits become fixed due to genetic drift in the populations that are capable of self-pollination. These facts lead us to one of the two possible inferences derived from our study that populations from the Indian Himalayas are relics of a once-widespread ancestral stock, which subsequently became fragmented during the course of evolution. Another inference might be that all the populations prevailing in the Indian Himalayas have originated from two types of genetic populations fixed due to natural selection a long time ago. This is also evident from the fact that in the cluster analysis, most of the individuals from the similar genetic population remained in the same group regardless of their geographical location. It also suggests that during the course of evolution, genotypes favoured by natural selection have been dispersed in the Himalayan region and have maintained themselves as constant, genetically similar lines for many generations. Although there is no distinct geographical barrier shown in the cluster analysis, six out of seven populations from the Pir Panjal range cluster into one group and three out of four populations from the Dhauladhar ranges cluster into another group. Out of four populations from the Garhwal Himalayas, two populations cluster into either group, as is the case of populations from the Greater Himalayan range which also cluster into both the groups.

### Gene flow between populations

Dispersal of pollens and migration of seeds determine the patterns of gene dispersion within and among populations after reproduction (Loveless and Hamrick 1984). The low levels of variance partitioned among populations suggest that a good level of gene flow is present among the populations of the Indian Himalayas, which is aptly confirmed by gene flow calculations (*N*_m_ = 2.13). The phenology of *P. hexandrum* suggests that pollen dispersal cannot be a factor accounting for the gene flow, which means that high gene flow is a result of seed dispersal. The fruits of *P. hexandrum* contain numerous seeds (80–120) **[see Supporting Information]**. A significant amount of gene flow was recorded across all the populations of the Indian Himalayas. The fruit is a berry which is not edible initially, but becomes edible as it ripens, and Himalayan birds and grazing animals feed on these fruits, thus facilitating seed dispersal (Rajkumar and Ahuja 2010). Further, the seed dispersal distance depends on various factors including the flight range of the birds and migration status of the grazers. All of the sites from which these populations were collected were situated in Himalayan regions that are accessed annually by numbers of tourists through the trekking trails of the Himalayas. Collectively, these short trails form a network known as “The Greater Himalayan Trail” which ranges from Nanga Parbat in Pakistan to Namcha Barwa in Tibet and includes the Himalayan mountains falling in the vicinity of Pakistan, India, Nepal, Bhutan and part of Tibet (Harris 1992; Choegyal 2011). In the cluster analysis, the population from the Sikkim region has been clustered along with the populations from the Pir Panjal range. The Sikkim population has been situated in the Kangchenjunga Himal section of the Greater Himalayas, which is located in the northeastern part of the Himalayas. This clustering suggests that these regions experience a significant amount of anthropogenic interference, and this activity also plays a major role in the dispersal of the germplasm along these trails, helping to increase the gene flow. High gene flow results in dampening of the local adaptation due to its homogenizing effect, which prevents population differentiation.

Furthermore, various reports suggest the unsustainable extraction of various medicinal plants from the Western Himalayas (Kala 2000, 2005; Uniyal *et al.* 2002; Kala and Farooquee 2004; Kala *et al.* 2006; Larsen and Olsen 2007; Larsen 2014). One such report has been published regarding the exploitation of *Picrorhiza kurroa*, another endangered medicinal plant (Uniyal *et al.* 2011) found in habitats like those of *P. hexandrum*. A similar kind of exploitation occurs for *P. hexandrum*. One can easily find seeds and roots of *P. hexandrum* by visiting the local healers and traditional medicinal practitioners at high elevations. Drug dealers follow the same Greater Himalayan Trail for trading of the raw material and drugs, also facilitating seed dispersal.

The number of seeds produced per plant is large, but the germination percentage is quite low (7–45 %). The seeds remain dormant for up to 3 years (Sreenivasulu *et al.* 2009). The production of a large number of seeds might be an adaptive strategy, so that a few, if not all, might germinate and establish new individuals, and as mentioned earlier, a solitary propagule is potent enough to produce a population if it is capable of self-pollination. This might have resulted in a population with the same genetic pool as that of the seed which was established after long-distance dispersal. The phenomenon of establishment of genetically similar populations following long-distance seed dispersal seems to have resulted in the two genetic populations present today.

## Conclusions

Based on the comprehensive molecular analysis of natural populations of *P. hexandrum,* it can be assumed that all the populations found in the Indian Himalayas are descendants of either one parent population from which two types of genotypes diverged or two different parent populations from different regions, whose dispersal to other regions was facilitated by humans, animals and birds. High variance partitioned within populations indicates that these populations are well sustained, but high levels of anthropogenic interference and habitat fragmentation are major threats for the sustainability of this plant in nature. Low levels of genetic diversity also pose a concern about the survival of the populations against ecological bottlenecks in the future. Moreover, stern conservation measures and laws need to be implemented urgently to limit the unauthorized uprooting and illegal trade of the rhizomes. The extent of overexploitation is such that during our frequent visits to the field, we found a significant amount of loss in the number of individuals within a season or two (e.g. between 2009 and 2011), the average number of plants per quadrat (1 m^2^) decreasing from 2 to 0.6 at Prashar (data not shown). If effective conservation measures are not undertaken soon, we are likely to lose the invaluable genetic resources of this important medicinal plant. Inferences derived from the current study will help to guide management and conservation policies. Moreover, high-throughput sequencing efforts are required to study environmental effects on the adaptation mechanism. This type of study will be extremely helpful in understanding the genes involved in divergent selection and local adaptation.

## Source of Funding

This research work was funded by the Council of Scientific and Industrial Research (CSIR), Government of India.

## Contributions by the Authors

R.K.S. and A.N. designed the study. A.N. collected the populations and conducted the AFLP and data analysis. P.S.A. helped in the coordination of the study. A.N. and R.K.S. wrote and approved the final version of the manuscript. All authors have read and approved the final manuscript.

## Conflicts of Interest Statement

None declared.

## Supporting Information

The following Supporting Information is available in the online version of this article –

**Table S1.** Analysis of molecular variance (AMOVA) between the different mountain ranges.

**Figure S1.** Representative AFLP profile of *P. hexandrum* samples revealed by E-ACA + M-CAG primer combination using from the automated DNA analyser (3730xl). (A) Lane window screen shot 1–96: Different *P. hexandrum* samples, (B) green fragments represent the detected fragments while red peaks are the marker fragments indicating size.

**Figure S2.** Pictorial representation of *P. hexandrum* plant in nature and its fruit showing numerous seeds in it.

**Figure S3.** Mantel test showing no correlation between genetic and geographic distance on the basis of AFLP data in 24 populations of *P. hexandrum*.

Additional Information
