# Supplementary material for: Genetic diversity of high-elevation populations of an endangered medicinal plant
Source: AoB Plants. 2014 Nov 21;7:plu076. doi: 10.1093/aobpla/plu076 (PMC4287688; doi:10.1093/aobpla/plu076)
Supplement: Additional Information [file supp_7_plu076_index.html]

Genetic diversity of high-elevation populations of an endangered medicinal plant — Genetic diversity of high-elevation populations of an endangered medicinal plant — Additional Information 

# Genetic diversity of high-elevation populations of an endangered medicinal plant

## Additional Information

Additional Information

**Files in this Data Supplement:**

- Additional Information - doc file
