# Supplementary material for: Genetic diversity of high-elevation populations of an endangered medicinal plant
Source: AoB Plants. 2014 Nov 21;7:plu076. doi: 10.1093/aobpla/plu076 (PMC4287688; doi:10.1093/aobpla/plu076)
Supplement: Additional Information [file supp_plu076_plu076supp.doc]

**Supplementary data**

Supplementary Table 1: AMOVA between the different mountain ranges

| Source | df | SS | MS | Est. Var. | % |
| --- | --- | --- | --- | --- | --- |
| Among ranges | 4 | 966.613 | 241.653 | 4.810 | 9% |
| Within ranges | 204 | 9822.200 | 48.148 | 48.148 | 91% |
| Total | 208 | 10788.813 |  | 52.958 | 100% |


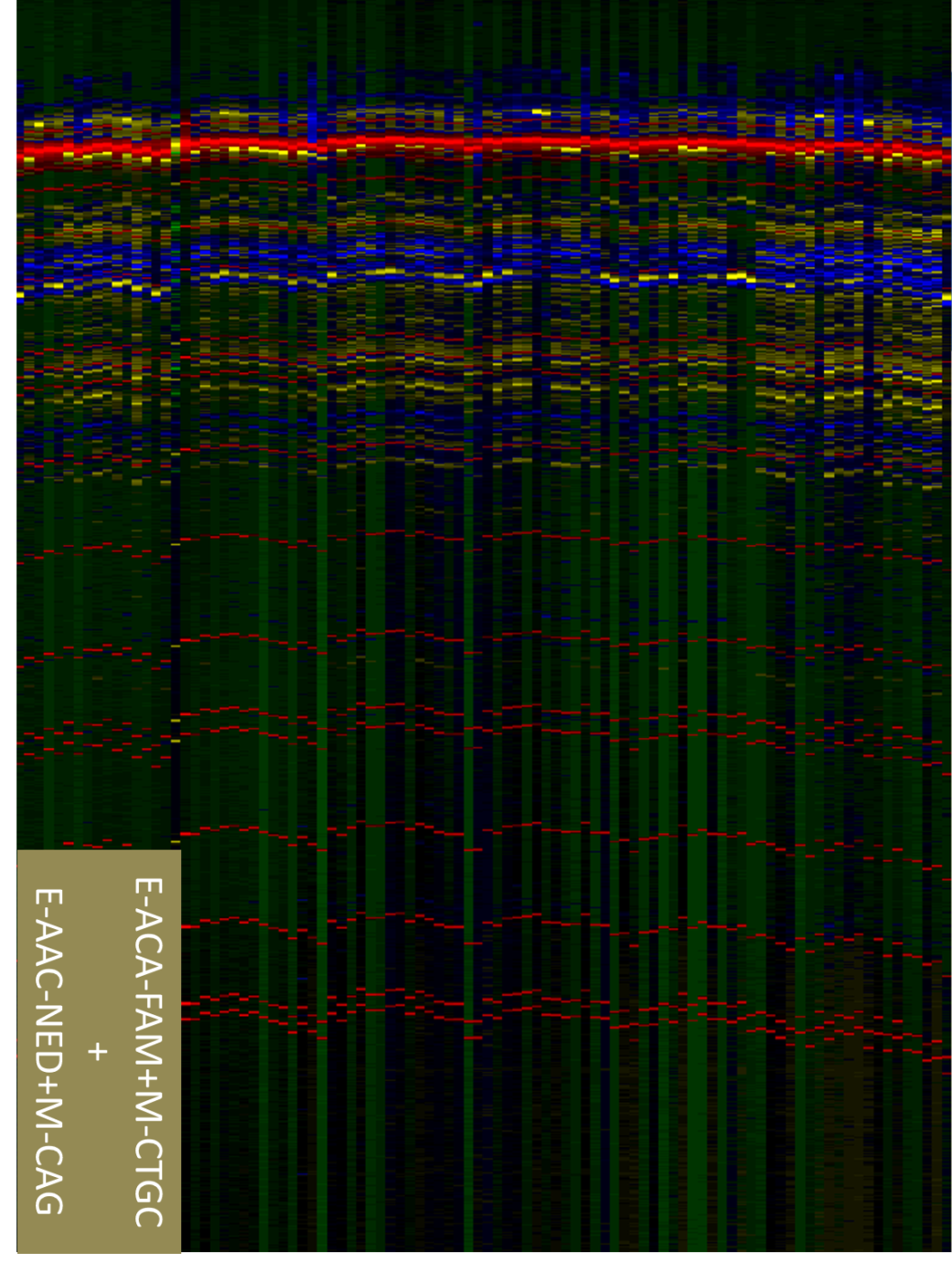


1 96

(A)


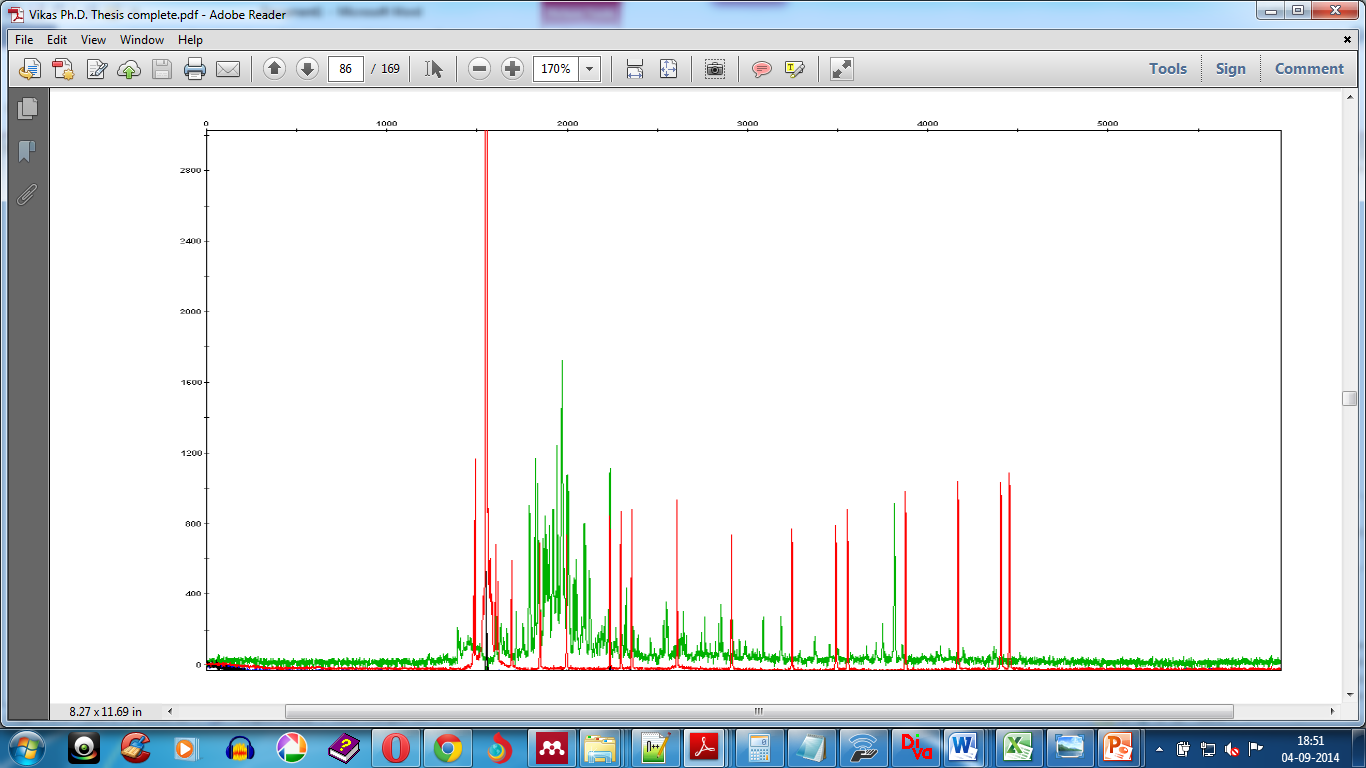
(B)

Supplementary Fig. 1. Representative AFLP profile of *P. hexandrum* samples revealed by E-ACA- +M-CAG primer combination using from automated DNA analyzer (3730xl). (A) Lane window screen shot 1 to 96: Different *P. hexandrum* samples , (B) Green fragments represents detected fragments while red peaks are the marker fragments indicating size


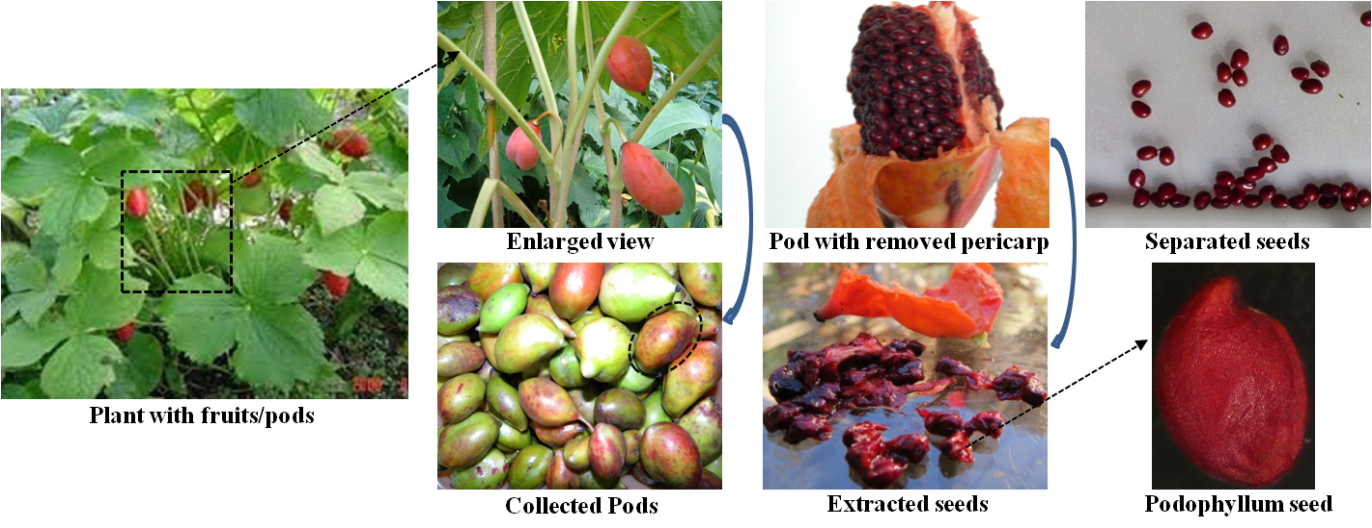


Supplementary Fig. 2 Pictorial representation of *P. hexandrum* plant in nature and its fruit showing numerous seeds in it


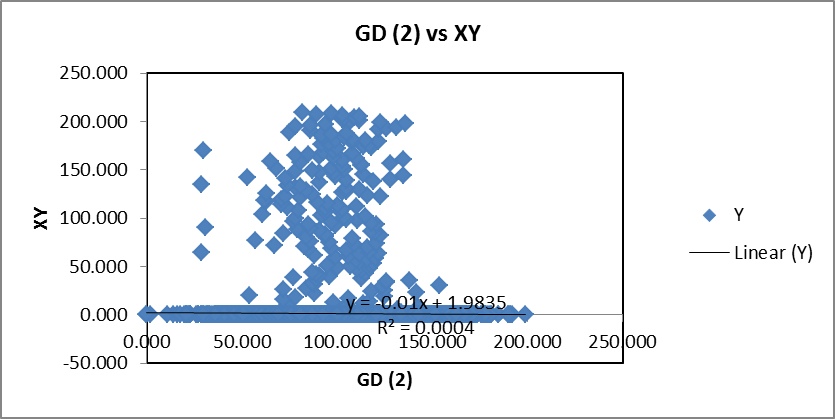


Supplementary Fig. 3 Mantel test showing no correlation between genetic and geographic distance on the basis of AFLP data in 24 populations of *P. hexandrum*
